# Temporal and spatial distribution of polycyclic aromatic hydrocarbons (PAHs) in the Danube River in Hungary

**DOI:** 10.1038/s41598-024-58793-2

**Published:** 2024-04-09

**Authors:** Ruqayah Ali Grmasha, Csilla Stenger-Kovács, Osamah J. Al-sareji, Raed A. Al-Juboori, Mónika Meiczinger, Manolia Andredaki, Ibijoke A. Idowu, Hasan Sh. Majdi, Khalid Hashim, Nadhir Al-Ansari

**Affiliations:** 1 Limnology Research Group, Center for Natural Science, University of Pannonia, Egyetem Utca 10, 8200 Veszprém, Hungary; 2 Environmental Research and Studies Center, University of Babylon, Al-Hillah, 51001 Iraq; 3 Sustainability Solutions Research Lab, Faculty of Engineering, University of Pannonia, Egyetem Str. 10, 8200 Veszprém, Hungary; 4 HUN-REN–PE Limnoecology Research Group, Egyetem Utca 10, 8200 Veszprém, Hungary; 5 NYUAD Water Research Center, New York University-Abu Dhabi Campus, Abu Dhabi, PO Box 129188, Abu Dhabi, United Arab Emirates; 6 Water and Environmental Engineering Research Group, Department of Built Environment, Aalto University, Aalto, PO Box 15200, 00076 Espoo, Finland; 7 School of Civil Engineering and Built Environment, Liverpool John Moores University, Liverpool, UK; 8 Department of Chemical Engineering and Petroleum Industries, Al‐Mustaqbal University College, Hillah, Iraq; 9 Dijlah University College, Baghdad, Iraq; 10 Department of Civil, Environmental and Natural Resources Engineering, Lulea University of Technology, Lulea, Sweden

**Keywords:** Danube River, Diagnostic ratios, Polycyclic aromatic hydrocarbons, Sediment, Water, Risk assessment, Environmental sciences, Engineering

## Abstract

The Danube is a significant transboundary river on a global scale, with several tributaries. The effluents from industrial operations and wastewater treatment plants have an impact on the river's aquatic ecosystem. These discharges provide a significant threat to aquatic life by deteriorating the quality of water and sediment. Hence, a total of 16 Polycyclic Aromatic Hydrocarbons (PAHs) compounds were analyzed at six locations along the river, covering a period of 12 months. The objective was to explore the temporal and spatial fluctuations of these chemicals in both water and sediment. The study revealed a significant fluctuation in the concentration of PAHs in water throughout the year, with levels ranging from 224.8 ng/L during the summer to 365.8 ng/L during the winter. Similarly, the concentration of PAHs in sediment samples varied from 316.7 ng/g in dry weight during the summer to 422.9 ng/g in dry weight during the winter. According to the Europe Drinking Water Directive, the levels of PAHs exceeded the permitted limit of 100 ng/L, resulting in a 124.8% rise in summer and a 265.8% increase in winter. The results suggest that the potential human-caused sources of PAHs were mostly derived from pyrolytic and pyrogenic processes, with pyrogenic sources being more dominant. Assessment of sediment quality standards (SQGs) showed that the levels of PAHs in sediments were below the Effect Range Low (ERL), except for acenaphthylene (Acy) and fluorene (Fl) concentrations. This suggests that there could be occasional biological consequences. The cumulative Individual Lifetime Cancer Risk (ILCR) exceeds 1/10^4^ for both adults and children in all sites.

## Introduction

Freshwater is a valuable and scarce resource for both individuals and ecosystems. The protection and preservation of the freshwater are increasingly important with the drive to promote food security and sustainable development growth agenda for life below water. The majority of the world's biggest cities were constructed on or near regions of freshwater, mostly rivers^1^. Most research on aquatic systems is primarily concerned with the effects of anthropogenic activities and natural phenomena, such as volcanism and biological processes, on both human health and ecology^2^. The emission levels of several anthropogenic toxins, including Polycyclic Aromatic Hydrocarbons (PAHs), have grown in the environment due to population expansion and associated increases in industrial, agricultural, and urban activities^3^. These chemicals have attracted significant worldwide interest due to their toxicity, persistence, bioaccumulation, and potential adverse health effects on living beings^4,5^. Thus, the United States Environmental Protection Agency (USEPA) and the European Union (EU) have identified 16 PAHs as priority pollutants among the hundreds of PAHs in the environment^6,7^. PAHs could be bounded to soil particles^8^ and could also adsorb on suspended particulate matter when entering the water and ultimately settle into the sediment. This is due to their high octanol–water partition coefficient and hydrophobic lipophilicity^9^. Consequently, river sediments are susceptible to PAHs accumulation and release. Moreover, they are commonly used as an indicator for detecting probable emission sources and determining the exposure risk of PAHs to benthic biotas^10^. Therefore, the environmental fate and the possible ecological risk related to PAHs are serious matters of public concern^11^. Numerous studies have employed water and sediment as important matrices for evaluating PAHs contamination in freshwater^12,13^.

The Water Framework Directive (WFD) obligates the European Union member states to achieve a satisfactory quantitative and qualitative assessment of the status of all bodies of water^14^. Nonetheless, some bodies of water have not yet reached this objective, and pollutants such as PAHs are not yet controlled^15^. Studies that have examined the PAHs level in the Danube River within the Hungarian regions can be categorized into two groups. The first group examined the upper area of the Danube River and the other group focused on the capital city (Budapest). Regarding the upper area, Nagy et al.^16^ investigated the PAHs concentrations and distribution in the surface water and bed sediments of the Hungarian upper section of the Danube River and in the Moson Danube branch during the period of 2001 to 2010. The study found that the concentrations of 16 PAHs in water samples ranged from 25.0 to 1208.0 ng/L, and in sediments ranged between 8.3 and 1202.5 ng/g in dry weight (dw). In another work, PAHs level in water from 2007 to 2010 ranged between 25.0 and 357.0 ng/L^17^. Moving to the second part, Visca et al.^18^ evaluated the PAHs contamination in the Danube River (only water samples) passing through Budapest in three locations within the city. The samplings performed in April 2017, November 2017, and October 2018. It should be mentioned; however, that the PAHs range is missing^18^. Caracciolo et al.^19^ also conducted a study to monitor the PAHs level in Budapest at three different points without, however, revealing the coordinates of those sampling points. Their measurement was for PAHs level in water in April and November 2017. The finding revealed that the total PAHs concentration ranged from 15.9 to 53.2 ng/L. According to the Joint Danube Survey 3 (JDS3) in 2015^20^, PAH concentrations in sediment (< 63 µm) along a 2581 km stretch of the Danube were analyzed on 65 sampling sites. The survey found that the maximum selected PAHs concentrations along the studied sties were 57, 690, 370, 489, 259, 328, and 179 µg/kg for anthracene, fluoranthene, benzo(a)pyrene, benzo(b)fluoranthene, benzo(k)fluoranthene, benzo(g,h,i)perylene and ndeno(1,2,3-cd)pyrene respectively. These records were found in Jochenstein, and Böfinger Halde in Germany. The high PAHs concentrations in the water column and sediment pore water downstream of Budapest, Hungary was observed also in a passive sampling campaign performed in 2013 in Joint Danube Survey 3^21,22^.

With a view to narrowing the gap in knowledge regarding PAHs concentration in the Danube River, the present investigation was designed to adequately investigate the spatial and temporal PAHs contamination of the river basin in Hungary. The main objectives are: (1) The spatial and temporal assessment of PAHs contamination in the Danube River; (2) The identification of the primary sources of PAHs contamination; (3) The evaluation of the eco-toxicological risk assessment of PAHs and; (4) The identification and qualification of the Incremental Lifetime Cancer Risk (ILCR), for both adults and children.

## Materials and methods

### Study area

**Figure 1:**
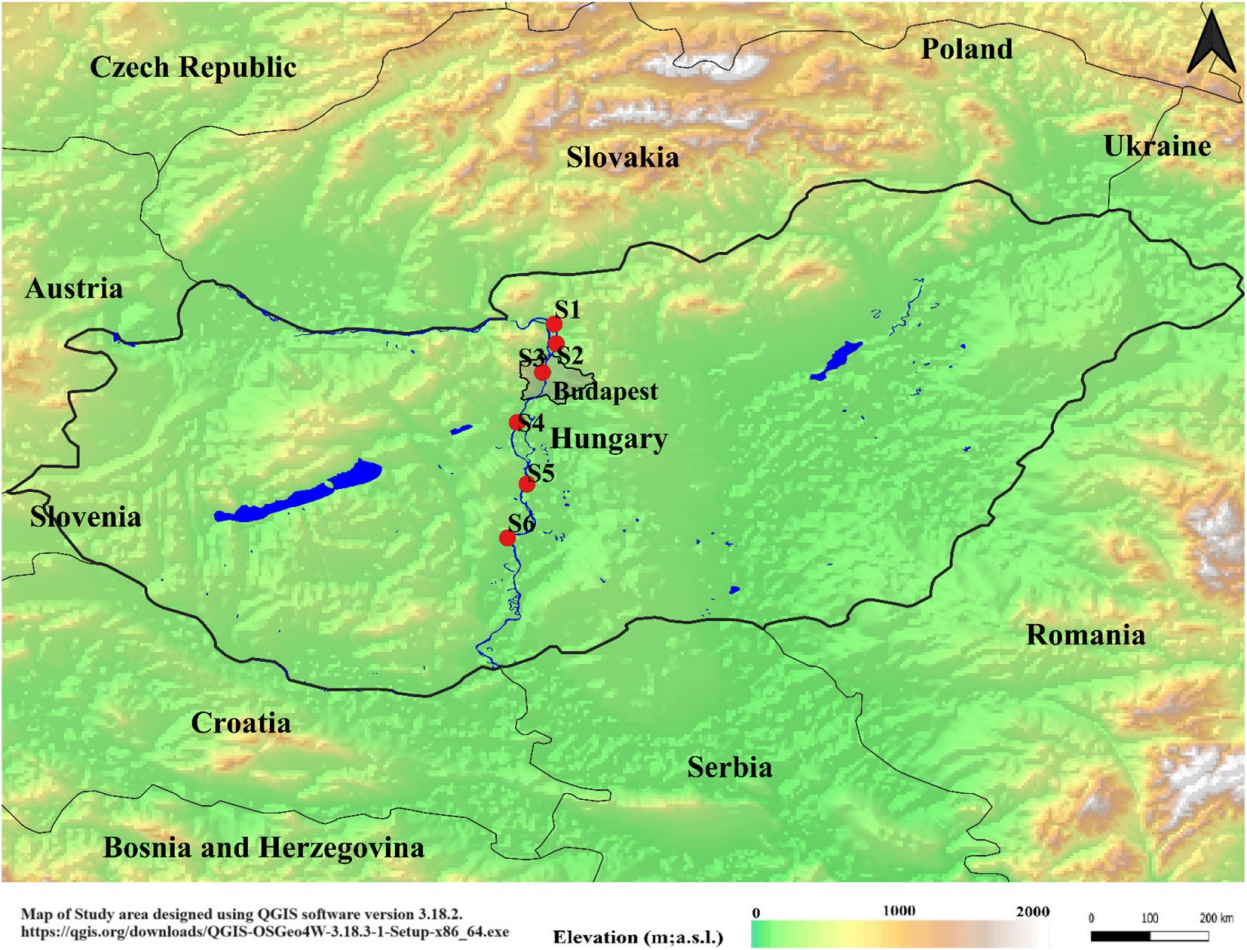
Sampling points in the Danube River within the Hungarian region.

With a length of 2780 km, the Danube is the second-longest river in Europe; its catchment area is 801,500 km^2^. The water of the Danube River Basin is used for a variety of reasons, including the production of drinking water, industrial and agricultural activity, leisure, hydroelectric power generation, and transportation. The length of the Hungarian Danube segment is 417 km (1850–1433 river kilometres (rkm)). Six locations were investigated along the proposed segment. Figure 1 depicts the sampling locations along the Danube River. Both S1 and S2 are representatives of the northern sites in our campaign in an area affected by both agricultural and industrial activities. Site 3 was taken at the heart of Budapest city, which happens to be situated downstream of a wastewater treatment plant, the second largest plant in the city (processing 180,000–200,000 m^3^/day of wastewater). Site 4 is located downstream of a polystyrene factory which is impact-resistant polystyrene and expandable polystyrene. Site 5 is located downstream of different industries, such as paper mill companies and electricity suppliers. Site 6 is located furthest south with respect to the other samples. This site was selected to be representative of the collective anthropogenic activities upstream. Table S1 shows the coordinates of the sampling sites.

### Chemicals

All solvents used were High-performance liquid chromatography (HPLC)-grade and acquired from Fisher Chemical Co. (USA), with a minimum purity of 99%. Supelco (Bellefonte, PA, USA) supplied the reference standards (QTM PAH-Mix, 2000 μg/mL) of the 16 PAHs. The 16 priority PAHs are including naphthalene (Nap), acenaphthylene (Acy), acenaphthene (Ace), fluorene (Fl), phenanthrene (Phe), anthracene (Ant), fluoranthene (Flu), pyrene (Pyr), benzo(a)anthracene (BaA), chrysene (Chr), benzo(b)fluoranthene (BbF), benzo(k)fluoranthene (BbF), Benzo (a) pyrene (BaP), Dibenz(a,h)anthracene (DBA), Benzo(ghi) perylene (BghiP), and Indeno (1, 2, 3-cd) pyrene (IND) were obtained from Supelco (Bellefonte, PA, USA). Sigma-Aldrich, USA, supplied solid-phase extraction membranes (ENVI™-18 DSK SPE Disk, diameter 47 mm), Sodium sulfate anhydrous, Silica gel (desiccant ~ 2–5 mm), as well as PAH recovery standards. Sodium Sulfate Anhydrous and Silica gel were put in a furnace (FI 600-60, Borel) at a temperature of 500 °C for 4 h to remove any moisture or organics before being transferred to a desiccator for storage until usage. In the experiments, Milli-Q water with a resistivity of 18.2 MΩ·cm at room temperature and a total organic carbon value of less than 5 ppb was utilized. Prior to each measurement, glassware was first cleaned using ultrasonic cleaners (Heidolph™, Fisher Scientific), washed afterwards with acetone, n-hexane, methanol, and dichloromethane to eliminate background pollutions and dried at 105 °C before use.

### Samples collection and pretreatment

The Danube has a mean discharge of 2350 m^3^/s at Budapest and 5600 m^3^/s at Belgrade^23,24^. The average depth of middle Danube region is 6 m to 10 m^25,26^. Throughout the study, the water and sediment (single) samples were collected on a monthly basis (i.e. two samples per month), from February 2022 to February 2023, over a 12-calendar month period. Sediments were often collected subsequent to water sampling in order to prevent disturbance and re-suspension of the sediment system into the water phase. Water samples were obtained at depths from 5 to 30 cm in 1 L brown glass containers that had been previously cleaned. The samples were temporarily kept in refrigerated containers with crushed ice until they were brought to the laboratory. After transporting the samples to the laboratory, they were filtered by glass fiber membrane of 0.45 μm^27^. Sediment samples were collected from the river bed at a depth 0–10 cm using a stainless-steel grab sampler and were then placed in clean polyethylene bags. Sediment samples were sieved through a 100-mesh sieve to remove any roots, debris, or large particles^28,29^. They were dried at 25 °C and were ground. Finally, they were dry-frozen (− 20 °C) until the time of further use.

### Extraction procedures of PAHs

#### Water sample extraction procedure

Solid phase extraction (SPE) was used to extract one litter of filtered water samples for the purpose of water sample extraction. The water sample extraction followed the previously reported procedures^27,30,31^. An SPE membrane was pre-washed using 6 mL of dichloromethane (DCM) before conditioning (activation) with 6 mL of methanol and 6 mL of ultrapure water, and 10 mL of methanol. A ten microliter of surrogate standard mixture solution (naphthalene-*d*_*8*_, anthracene-*d*_*10*_, fluoranthene-*d*_*10*_, perylene-*d*_*12*_) was added to one litter of the water sample. This step was to enrich the sample, which was then passed through the SPE at a flow rate of 3 mL/min. After the extraction was finished, a vacuum pump was utilized to dry the column, and 6 mL of dichloromethane was added to soak it for 5 min before elution into a clean glass test tube. By using nitrogen, the eluate was concentrated to 0.5 mL prior to the addition of ten microliter of standard mixture solution. Then the samples were analyzed using gas chromatography-mass spectrometry (GC–MS) analysis.

#### Sediment sample extraction procedure

The freeze-dried sediment samples were pulverized using a mortar and pestle and were sieved using a 100-mesh to remove large particles. Sediment samples dried and homogenized were put into brown glass vials for further laboratory analysis. The sediment sample extraction followed the previously reported procedures^29–31^. A sample of 2 g was precisely weighed out. Five milliliters of acetone/n-hexane (1:1, v/v) and a standard surrogate solution were added to the test tube. Afterwards, they were vortexed for 60 s, followed by 15 min of ultrasonic extraction in a water bath. The test tubes were then centrifuged for 20 min at 2000 rpm to separate the solid and liquid phases. Using a Pasteur pipette, the supernatant was transferred into another clean test tube. Then, 5 mL of a 1:1 mixture of acetone and n-hexane was added to each sample. In one test tube, the extracts were mixed, and activated copper was added for desulfurization. Then, sodium sulfate anhydrous was applied to eliminate water, followed by a concentration step to 0.5 mL using a nitrogen-blowing concentrator. Finally, an internal standard solution was added for the GC–MS analysis. PAHs were measured using GC–MS (gas chromatography mass spectrometry) type Agilent 6890 N 5975C mass selective detector, Agilent Technologies, USA. Helium was utilized as a carrier gas at 1.5 mL/min using an HP-5MS gas chromatography column (30 m 0.32 mm 0.25 µm)^8^. The selective ion scanning (SIM) mode was utilized for quantitative analysis. The injector temperature was set to 300 °C. The oven temperature was programmed as follows: the initial temperature was set to 100 °C for one minute, after which it increased to 300 °C at a rate of 8 °C per minute and remained at 300 °C for 39 min. Triplicate measurements were recorded for each sample resulting in a relative standard deviation of less than 10.2%.

#### QC/QA

In this study, the quality controls included triplicate samples, matrix spike standards, calibration standards, a procedural blank, and detection limits. Prior to each measurement, the glassware was first cleaned using ultrasonic cleaners, washed afterwards with acetone, n-hexane, methanol, and dichloromethane to eliminate background pollution and dried at 105 °C before use. Utilizing the dry weight approach, the concentration of 16 PAHs in sediment samples was determined. The lowest detection limits (LOD) were determined using analyte concentration and a threefold signal-to-noise ratio^32,33^. The range of LOD for water was between 0.02 and 0.59 ng/L, while for sediment was between 0.37 and 0.96 ng/g in dw. PAHs recovery was determined by spiking water and sediment samples with standard solutions. A procedure blank (solvent), a spiked blank (standards added to solvent), and sample triplicates were conducted for each sample. Analysis of method blanks proved the absence of detectable PAH contamination. The recovery ranges for 16 PAHs in water, and sediment samples were 91.5% ± 4.3% to 104.1% ± 7.6% and 86.8% ± 5.5% to 99.2% ± 6.2%, respectively. For water samples, the recovery ranges of the spiking standards were 92.8% ± 4.5% to 109.7% ± 6.9%, and for sediment samples, they were 90.5% ± 8.3% to 97.4% ± 4.4%. For recovery, the concentrations of 16 PAHs were adjusted. Reference and blank samples were measured to confirm the accuracy of the analysis. Moreover, each sample was measured three times, resulting in a relative standard deviation between 1.7 and 10.4%, which is within the acceptable limit (< 25%). Mean values are presented for the measurements. The data in this study were tested to the Kolmogorov–Smirnov normality at a significance level of 0.05.

### Eco-toxicological concerns and Incremental Lifetime Cancer Risk (ILCR) for sediment

The sediment sample assessment for ecological risk followed the methodology outlined by^34^. PAHs levels in sediments were assessed according to sediment quality standards (SQGs)^35^. Compared with the concentration for each PAH to the Effect Range Low (ERL) and Effect Range Median (ERM) values, the ecological risk to aquatic species posed by contact with sediment-bound PAHs was ascertained. The SQGs involve three classifications of chemical concentrations that define the levels of adverse chemical effects on biology: (1) minimal effects range with rare biological effects (< ERL), (2) possible effects range with occasional biological effects (≥ ERL and < ERM), and (3) probable effects range with frequent biological effects (≥ ERM).

For the purpose of comparing the carcinogenicity of PAHs to that of BaP, the toxic equivalency factor (TEF) approach was employed to determine the BaP equivalence (BaPeq) of PAHs^36^. Due to its high carcinogenicity, BaP has been chosen as a reference chemical in the TEF estimates and assigned a value of one so that the carcinogenicity of each PAH can be estimated relative to BaP. The recorded TEF values are shown in Table S2. Based on their relative carcinogenicity to BaP, individual PAHs have unique TEF numbers. The formulas listed below are utilized to determine the toxic equivalent quotient (TEQ) for every location in the present investigation.

1 $${{\text{BaPeq}}}_{\mathrm{i }}= ({{\text{PAH}}}_{\mathrm{i }}\times {{\text{TEF}}}_{\mathrm{i }})$$

2 $${\text{TEQ}}={\sum }_{1}^{{\text{n}}}({{\text{PAH}}}_{\mathrm{i }}\times {{\text{TEF}}}_{\mathrm{i }})$$

where PAHi is the PAH concentration and TEFi is the toxic equivalency factor.

Using the USEPA's ILCR model, which examined the three main routes of exposure to contaminants (ingestion, dermal contact, and inhalation), a risk assessment to PAHs in river sediments was performed. This assessment was required because of people's daily reliance on the region's aquatic resources^37^. ILCR is used to estimate the human cancer risk posed by exposure to environmental PAHs. The overall carcinogenic risk was determined by summing the hazards associated with the three routes of exposure. Table S3 and Eqs. (3, 4, 5, and 6) respectively explain the ILCR assessment parameters and model formulations^37–39^.

3 $${{\text{ILCR}}}_{{\text{ingestion}}}={\text{CS}}\times {{\text{IR}}}_{{\text{ingestion}}}\times {\text{EF}}\times {\text{ED}}\times \left({{\text{CSF}}}_{{\text{ingestion}}}\times \sqrt[3]{\frac{{\text{BW}}}{70}}\right)\times {\left({\text{BW}}\times {\text{AT}}\times {10}^{6}\right)}^{-1}$$

4 $${{\text{ILCR}}}_{{\text{inhalation}}}={\text{CS}}\times {{\text{IR}}}_{{\text{inhalation}}} \times {\text{EF}}\times {\text{ED}}\times \left({{\text{CSF}}}_{{\text{inhalation}}}\times \sqrt[3]{\frac{{\text{BW}}}{70}}\right)\times ({{\text{BW}}\times {\text{AT}}\times {\text{PEF}})}^{-1}$$

5 $${{\text{ILCR}}}_{\mathrm{dermal \,contact}}={\text{CS}}\times {\text{SA}}\times {\text{AF}}\times {\text{ABS}}\times {\text{EF}}\times {\text{ED}}\times \left({{\text{CSF}}}_{\mathrm{dermal \,contact}}\times \sqrt[3]{\frac{{\text{BW}}}{70}}\right)\times ({{\text{BW}}\times {\text{AT}}\times {10}^{6})}^{-1}$$

6 $$\mathrm{Carcinogenic \,risk }= {{{\text{ILCR}}}_{{\text{ingestion}}}+\mathrm{ ILCR}}_{\mathrm{dermal \,contact}}+ {{\text{ILCR}}}_{{\text{inhalation}}}$$

CSF is the carcinogenic slope factor, which is represented in units of (mg kg^−1^ day^−1^)^−1^. According to the USEPA, the CSF concentrations of BaP for the three exposure pathways^40^ are 25, 7, 3 and 3.85 mg/kg/day^−1^. CS is the total PAHs concentrations that transformed to hazardous equivalents of BaP using the Toxic Equivalence Factor (TEF) (in ng/g). Calculation of the ILCR relies heavily on the detection of PAHs as BaP-equivalent concentrations using the TEF of each PAHs relative to BaP. The total ILCR is equal to the sum of three routes: skin contact, oral consumption, and inhalation. If the ILCR is less than 1/10^6^, it is deemed inconsequential; if it is more than 1/10^4^, there is a reason for serious worry^41^.

### Ethics approval

All authors have read, understood, and complied as applicable with the statement on “Ethical responsibilities of Authors” as found in the Instructions for Authors.

### Statement

This research was part of a doctoral thesis for Ruqayah Ali Grmasha.

## Results and discussion

### PAHs spatial distribution and seasonal variation in water and sediment

#### Water

**Figure 2:**
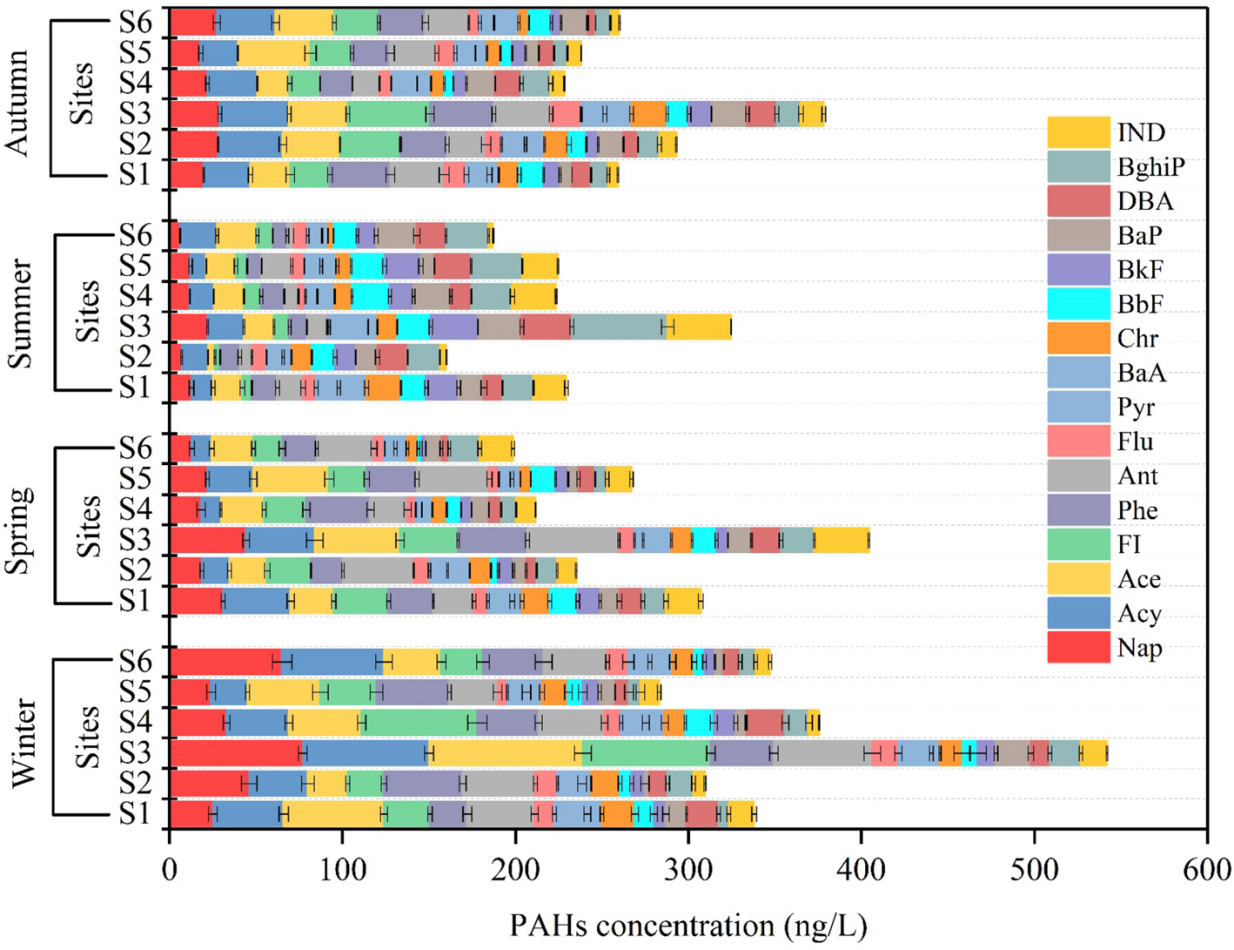
PAHs concentration in water in the six locations with respect to seasons.

**Figure 3:**
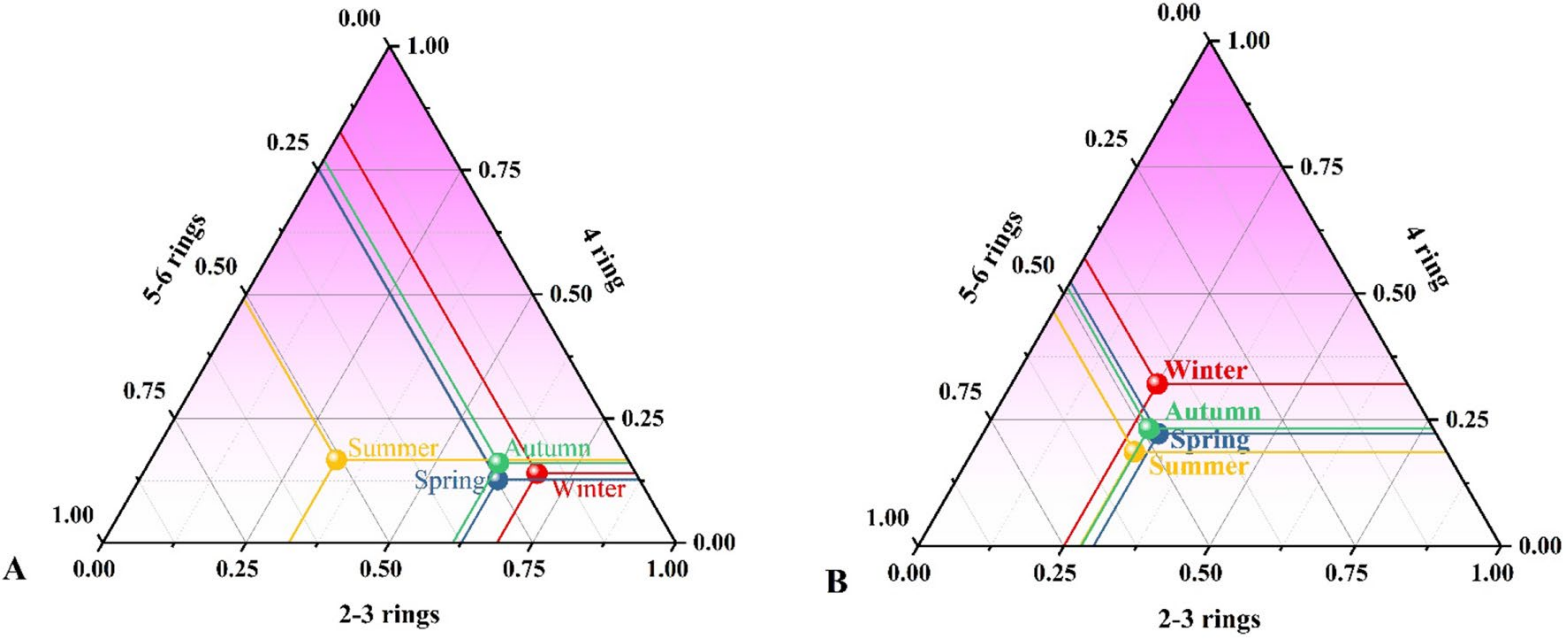
PAHs rings percentages in (**A**) water and (**B**) sediment among different sampling seasons: winter, spring, summer, and autumn.

Figure 2 shows the 16 identified PAHs concentrations in the water of the Danube River. The total PAHs contents in water for all seasons were ranging from 224.8 (in summer) to 365.8 ng/L (in winter). The seasonal concentration variation of PAHs was 283.1–541.9 ng/L in winter, 198.6–404.3 ng/L in spring, 160.0–324.6 ng/L in summer, and 228.2–378.1 ng/L in autumn. The concentrations of LMWPAHs (low molecular weight PAHs, 2–3 rings): Nap, Acy, Ace, Fl, Phe, and Ant) in water samples of the Danube River were greater than the concentrations of HMWPAHs (high molecular weight PAHs 4–6 rings): Flu, Pyr, BaA, Chr, BaP, DBA, BkF, BbF, IND and BghiP, and in all four seasons. The HMWPAHs recorded the highest values during the summer season (Fig. 2). In Fig. 3, the PAHs profile in water and sediment is presented. Ace was found to be the most predominant component, with levels between 16 and 47.8 ng/L, followed by Nap, Acy, FI, Ant, and Phe with values between 11.9 and 45.0 ng/L, 15.5 and 44.0 ng/L, 7.1 and 40.9 ng/L, 10.5 and 40.2 ng/L, and 11.0 and 36.0 ng/L, respectively. The highest HMWPAHs in water samples were BghiP (11.4–28.3 ng/L), IND (8.2–17.9 ng/L), DBA (9.6–17.8 ng/L), BaP (9.1–17.5 ng/L), BkF (7.2–17.2 ng/L), BbF (9.5–17.1 ng/L), Pyr (7.8–14.9 ng/L), Chr (10.1–14.7 ng/L), Flu (5.9–11.0 ng/L), and BaA (8.2–10.1 ng/L). The concentrations of ∑PAHs in the water of the Danube River were greater in the winter, spring, and autumn seasons compared to the summer season, which was attributed to the lower temperatures during these sampling times. The variation in the patterns of PAHs in water throughout various sample periods (Fig. 2) is likely related to connected with the PAHs molecular weight and degradation, which is more evident during warm seasons^42^. In contrast to HMWPAHs (4–6 rings) which have a poor water solubility and dissolution rate and are, hence, more resistant to decomposition, LMWPAHs (2–3 ring PAHs) are more degradable and soluble during warm seasons^43^. The 2–3 rings PAHs are higher in cold seasons, and 4–6 rings PAHs are higher in the hot season (Fig. 3); this is because of the reduction in the content of LMWPAHs in water. Furthermore, the current data reveal that PAHs concentration in water also varies across the sampling sites. The highest concentrations of PAHs in water were found at the S3 (the center of Budapest) sampling location during cold seasons (Fig. 2), in conjunction with high amounts of LMWPAHs, indicating a local origin of recently generated LMWPAHs resulting from atmospheric deposition caused by vehicular and industrial emissions from various plants. Furthermore, it was revealed that the sediments of S5 had significant quantities of PAHs throughout the winter and autumn seasons. The presence of PAHs in water samples was also examined by Visca and his colleagues^18^. A significant amount of PAHs were found in the investigation, with the highest records occurring in April. The PAHs were sourced from home heating, automobile emissions, air transport dynamics, and the wastewater treatment facility that was located nearby.

The ∑PAHs concentrations in the water samples in all seasons (ranges from the lowest of 224.8 in summer to the highest of 365.8 ng/L in winter) are higher than those found in the Danube River and tributaries (67.0–96.0 ng/L) from the territory of Hungary^17,44^, Danube River (16.0–133.0 ng/L)^45^, Danube River measurements (15.9–53.2 ng/L)^19^. Within the European Union, specifically under the Drinking Water Directive, the permissible concentration of PAHs in water is defined at a maximum total level of 100 ng/L^46,47^. The ∑PAHs levels within the seasons were higher than the standard limits specified by WFD by 124.8% and 265.8% increase in summer and winter respectively.

Contrarily, the ∑PAHs concentrations are lower than those found in the Raba River (the largest Danube tributary in Hungary), with a range between 41.0–437.0 ng/L^48^. These variations are likely due to the difference in the selected sampling sites. This study deliberately selected sites where the potential source of contamination lies ahead. Generally, these concentrations are also lower than those found in other countries such as the Tiber River in Italy, Humen River, Bai Chao and Chaobai Rivers in China, with PAHs range of 10.3–951.6 ng/L, 311.1–1012.8 ng/L, and 55.0–882.0 ng/L, respectively^49–51^. In addition, it was similar to those reported in China by Chen et al.^52^ in the Yinma River (23.2–386.9 ng/L).

#### Sediment

**Figure 4:**
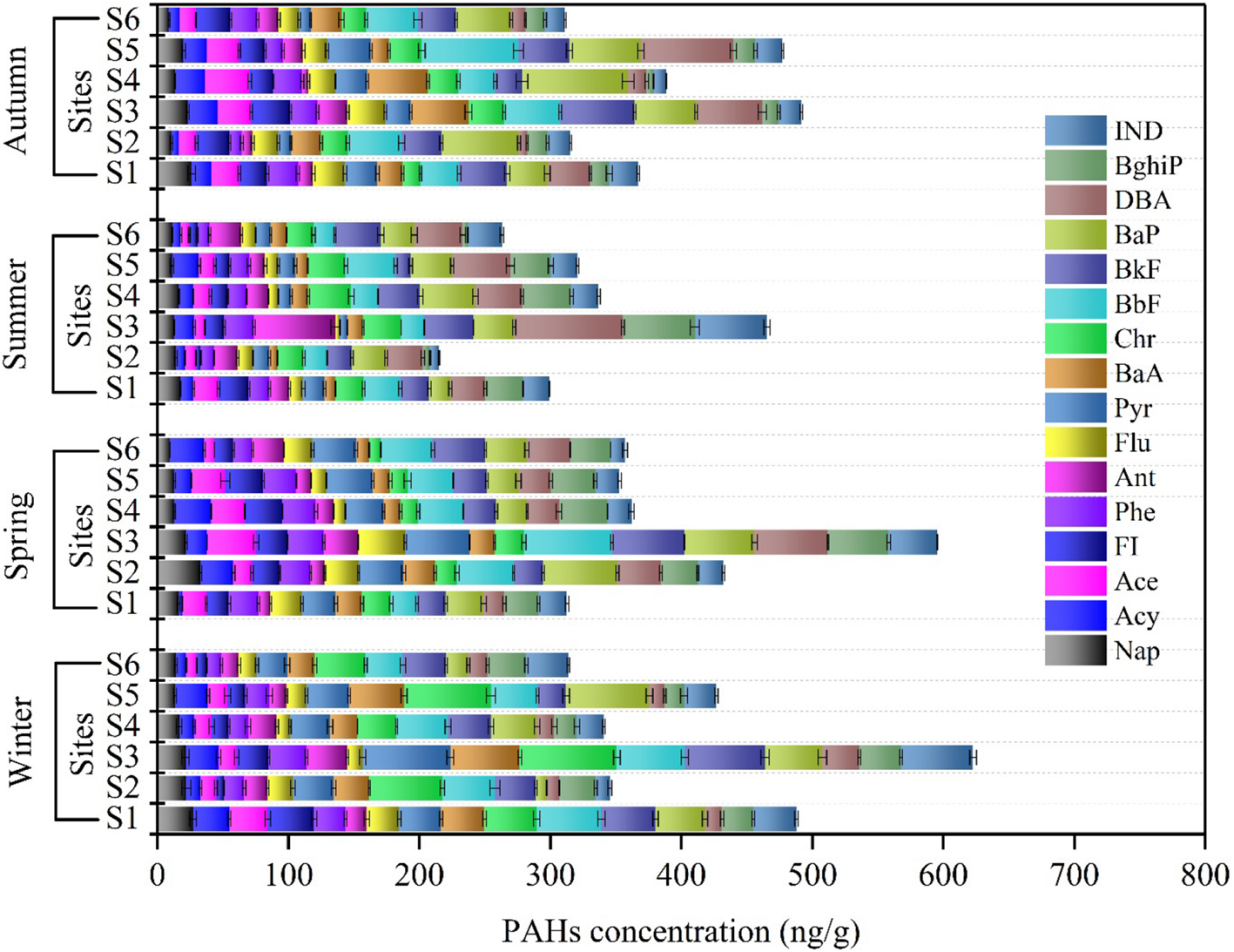
PAHs concentration in sediment samples among various sampling sites and seasons campaigns.

The concentrations of PAHs in sediments are presented in Fig. 4. PAHs concentrations in all four seasons for the sediment samples were ranging from 316.7 in summer to 422.9 ng/g dw in winter. The concentration variation of PAHs within seasons is as follows: 313.7–622.7 ng/g in winter, 312.7–595.4 ng/g in spring, 215.1–465.4 ng/g in summer, and 311.3–491.7 ng/g in autumn. The variance of PAHs in the sediments of the Danube River indicates that PAHs levels vary with sampling sites, indicating the anthropogenic sources along the Danube River, as well as with seasons, given that the samples were gathered at various periods and places. Contrarily to the reported concentrations of PAHs in water, HMWPAHs are more prevalent than LMWPAHs in sediments of the Danube River (Fig. 3), which is in accordance with other studies^53,54^. LMWPAHs had greater water solubility owing to lower octanol–water coefficients and were more volatile than HMWPAHs, which makes them record the lowest concentrations values in sediment samples^55^. In contrast, HMWPAHs have low water solubility, greater partitioning coefficients, and high hydrophobicity in aqueous conditions^56^. BaP, Chr, BbF, and BkF are the most predominant PAHs in sediments samples, with records ranging between 28.7 and 52.4 ng/g, 16.1 and 50.8 ng/g, 22.9 and 42.6 ng/g, as well as 14.9 and 42.4 ng/g, respectively-followed by BkF, Pyr, BghiP, and BaA with values between 26.3–36.8 ng/g, 11.4–36.0 ng/g 13.3–33.8 ng/g, and 10.1–32.8 ng/g, respectively. Consistent with water samples, S3 recorded the highest PAH concentration (Fig. 4). IND and Flu had the lowest concentrations with values ranging from 17.0–29.3 ng/g and 9.0–21.4 ng/g, respectively. LMWPAHs that had the highest concentrations are Ant (13.2–24.8 ng/g), FI (11.9–23.3 ng/g), and Phe (14.5–23.2 ng/g), while Acy, Nap, and Ace scored the lowest concentration ranges of 11.6–18.4 ng/g, 14.0–19.2 ng/g, and 11.2–22.0 ng/g, respectively. No distinctive patterns were identified for PAHs in sediments samples during the different seasons indicating the sediments independence of temporal variations. Sediment pollution assessed by total PAHs concentrations may be categorized as follows: (A) low polluted (less than 100 ng/g), (B) moderately polluted (between 101 and 1000 ng/g), (C) highly polluted (between 1001 and 5000 ng/g), and (D) very polluted (more than 5000 ng/g)^57^. Consequently, the pollution levels of the sediments from the Danube River can be categorized as low pollution.

**Figure 5:**
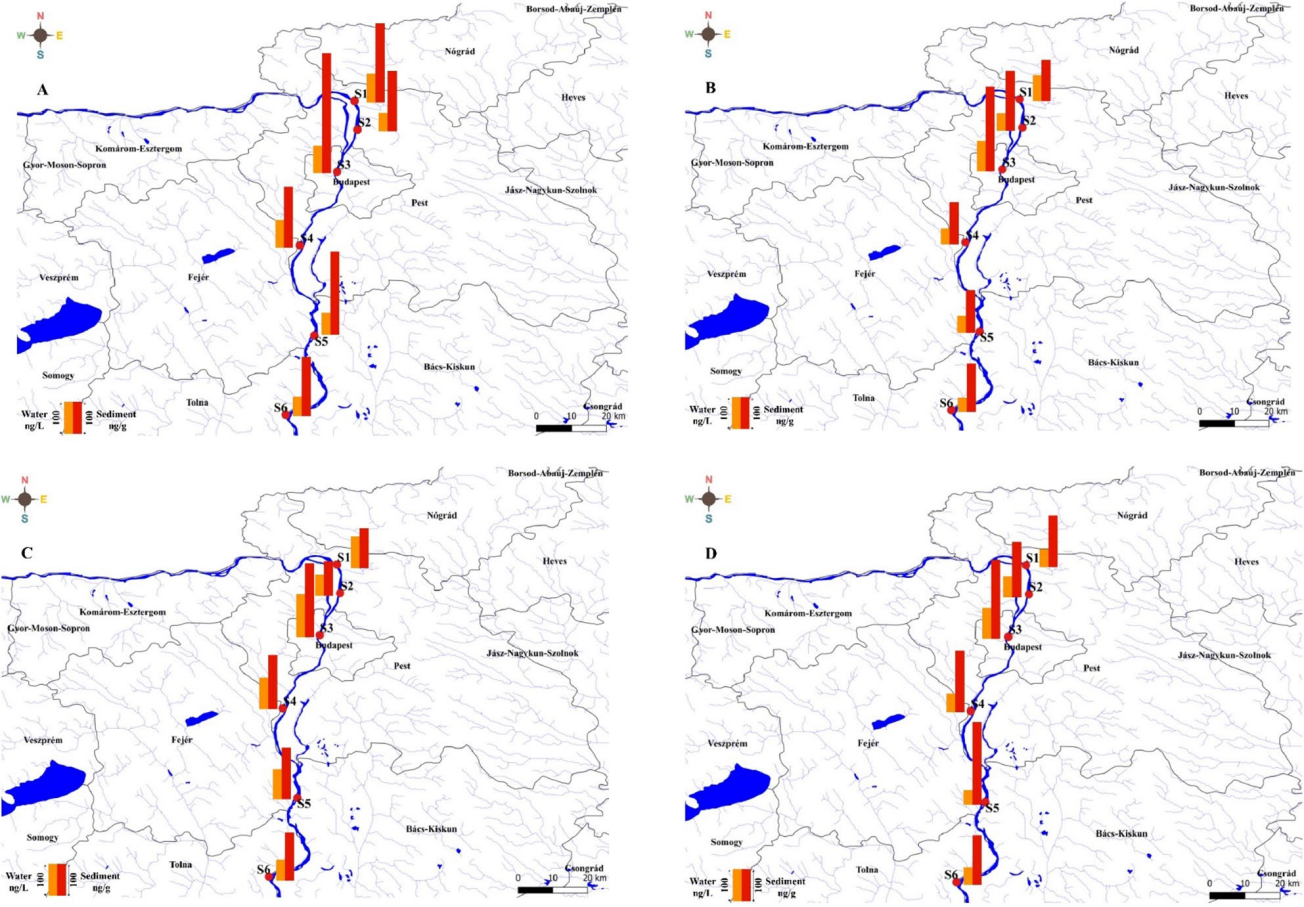
7CPAHs for each site for winter (**A**), spring (**B**), summer (**C**), and autumn (**D**). Map of study area designed using QGIS software version 3.18.2. https://qgis.org/downloads/QGIS-OSGeo4W-3.18.3-1-Setup-x86_64.exe.

The seven carcinogenic PAHs (CPAHs) account for most of the total PAHs ranging from 181.09 in summer to 240.0 ng/g in winter, with the CPAHs exhibiting the same spatial distribution as ΣPAHs in S3. Figure 5 shows the 7CPAHs distribution in water and sediment along the Danube River in all seasons. Variations in the spatial distribution of PAH concentrations in sediments can be attributed to various factors^3,53,58,59^, such as (1) discharge of untreated municipal wastewater, traffic emissions, industrial activities, and fuel consumption; (2) various hydrodynamic systems related to meteorological conditions that can stimulate resuspension and re-deposition of the sediments; (3) alteration in sediment textural characteristics based on spatial properties of sampling sites and (4) the presence of redox conditions in sediments and PAH biodegradation. The PAHs maximum concentration in site 3 sampling locations can be due to the position of S3 inside Budapest and downstream of the wastewater treatment plant. The PAHs concentrations in wastewater are a significant cause of concern for the industry. PAHs are regarded as hazardous components because of their highly toxic and polluting possibilities, which can persist for many decades in the environment, and their carcinogenic, genotoxic, and mutagenic effects, which can cause irreparable harm to individuals' health^60,61^. PAHs pollution can occur in wastewater effluent when they are not eliminated, and as a result, they can enter river water from these sources^62–64^.

The concentrations of PAHs in sediment samples from the Danube River (316.7 in summer − 422.9 ng/g, dw in winter) were higher than those detected in the Hungarian upper section of the Danube River and its tributaries (35.2–288.3 ng/g)^44^ and Danube River and Moson Danube Arm (Hungary) (118.0–283.0 ng/g)^45^ but lower than those reported in Hungarian upper section of the Danube River and the Moson Danube branch (8.3–1202.5 ng/g)^48^. Generally, the measured PAHs concentrations in the present study were congruent with literature from other country such as Soltan Abad River, Iran (180.3–504.0 ng/g)^65^ as well as Ovia River, Nigeria (5.2–573.3 ng/g)^66^. Followed by the lower comparison to the concentrations that were reported by Liu et al*.*^67^, in Rivers in Shanghai, China (248.8–36,198.2 ng/g). PAHs concentrations in soils of the Seine River basin, France ranged from 450 to 5650 µg/kg^68^. Otte and co-worker found that the 16PAHs in the sediments of Elbe River Estuary, Germany was moderately contaminated with PAHs ranged from 0.02 to 0.906 mg/g dw^69^. PAHs in water and sediments from Tiber River and estuary, Italy were ranged from 10.3 to 951.6 ng/L and from 36.2 to 545.6 ng/g in water and in sediment samples, respectively^50^. The PAHs level in Sele River in South of Italy showed that the total PAH concentration ranged from 632.4 to 844.9 ng/g dw. Furthermore, the PAHs in Sarno and Volturno River sediments were in the range of 5.2–678.6 ng/g and 434.8–872.1 ng/g respectively^70^.

### Sources identification ratios of PAHs in water and sediment samples

Based on the average concentrations of individual PAHs in water and sediments for each sampling season (winter, spring, summer, and autumn), the following diagnostic ratios were determined to identify the dominant sources and the related emission routes. The precise meaning of each ratio related to Flu/(Flu + Pyr), LMW/HMW, Flu/Pyr, BaA/(BaA + Chr), IND/(IND + BghiP), IND/BghiP, and BaA/(BaA + Chr) with the determined ratios are given in Tables S4 and S5 for water and sediments, respectively. According to the literature, a ratio of BaA/(BaA + Chr) less than 0.2 suggests that PAHs are mostly generated from petrogenic inputs (liquid fuel discharges), a ratio between 0.2 and 0.35 shows that PAHs are sourced from mixed sources (petrogenic/pyrogenic), and a ratio greater than 0.35 indicates that PAHs are primarily formed from pyrogenic—combustion of solid fuel—natural sources such as biomass and coal. A ratio of Flu/ (Flu + Pyr) less than 0.4 indicates that PAHs originate from petrogenic inputs, a ratio between 0.4 and 0.5 indicates that they are derived from pyrolytic (burning of liquid fossil fuels and crude oil, vehicles), and a ratio greater than 0.5 indicates that they are sourced from pyrogenic—combustion of solid fuel. A ratio of IND/ (IND + BghiP) lower than 0.2 indicates that PAHs originate from petrogenic inputs, a ratio between 0.2 and 0.5 indicates that they are derived from pyrolytic sources, and a ratio greater than 0.5 indicates that they are sourced from pyrogenic sources.

**Figure 6:**
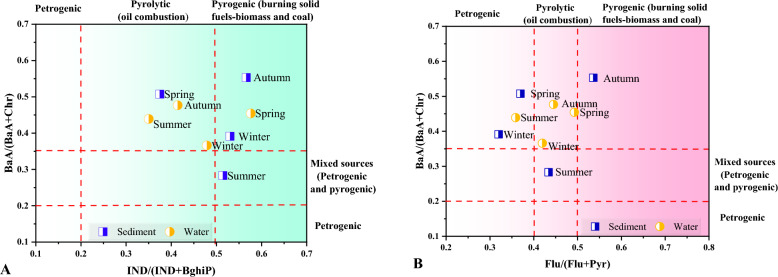
Cross plots of the diagnostic ratios of (**A**) BaA/(BaA + Chr) to IND/ (IND + BghiP) and (**B**) ratios BaA/(BaA + Chr) to Flu/(Flu + Pyr) for water and sediment samples in all seasons from the Danube River.

The ratios plot of BaA/(BaA + Chr) against Flu/(Flu + Pyr) and ratios of BaA/(BaA + Chr) against IND/(IND + BghiP) are presented in Fig. 6. Generally, LMWPAHs originate from oil or fuel spills and have a short lifetime in the ecosystem, whereas HMWPAHs arise from combustion products, pyrolysis, or petrogenic origins^3^. As it is shown in Fig. 6, Flu/(Flu + Pyr) ratios are between 0.4–0.5 in all seasons except for summer for water samples, while the opposite was observed in sediment samples, indicating pyrolytic sources in both cases. Furthermore, these ratios were < 0.4 in the summer season for water samples and in both the winter and spring seasons for sediment samples indicating petrogenic inputs. The only time these ratios > 0.5 were in autumn for sediment samples which explains that wood or coal, and grass combustion are the main PAHs origins.

The BaA/(BaA + Chr) ratios for both water and sediment samples were more than 0.35, indicating pyrogenic sources, except for the summer season in sediments which are scattered and clustered between 0.2 and 0.35, indicating mixed sources (petrogenic/pyrogenic). The ratios of IND/(IND + BghiP) for water samples were more than 0.5 in the spring season only and for all four seasons, except for spring, for sediments samples, suggesting the contribution of combustion of solid fossil fuel-like biomass and coal in agricultural regions. The ratios of IND/(IND + BghiP) were between 0.2 and 0.5 in the winter, summer, and autumn seasons for water samples and in the spring season for sediments samples indicating pyrolytic sources. The BaP/BghiP ratio in both water and sediment sampling was more than 0.6 in all sampling seasons, indicating PAHs of petrogenic origin. Overall results indicate, the putative anthropogenic sources of PAHs were verified to be both pyrolytic (incomplete combustion of liquid fossil fuels and vehicle exhaust emissions) and pyrogenic (incomplete combustion of biomass and coal), with pyrogenic sources predominating over pyrolytic sources.

### Principal component analysis (PCA) based on PAHs in water and sediment samples

**Figure 7:**
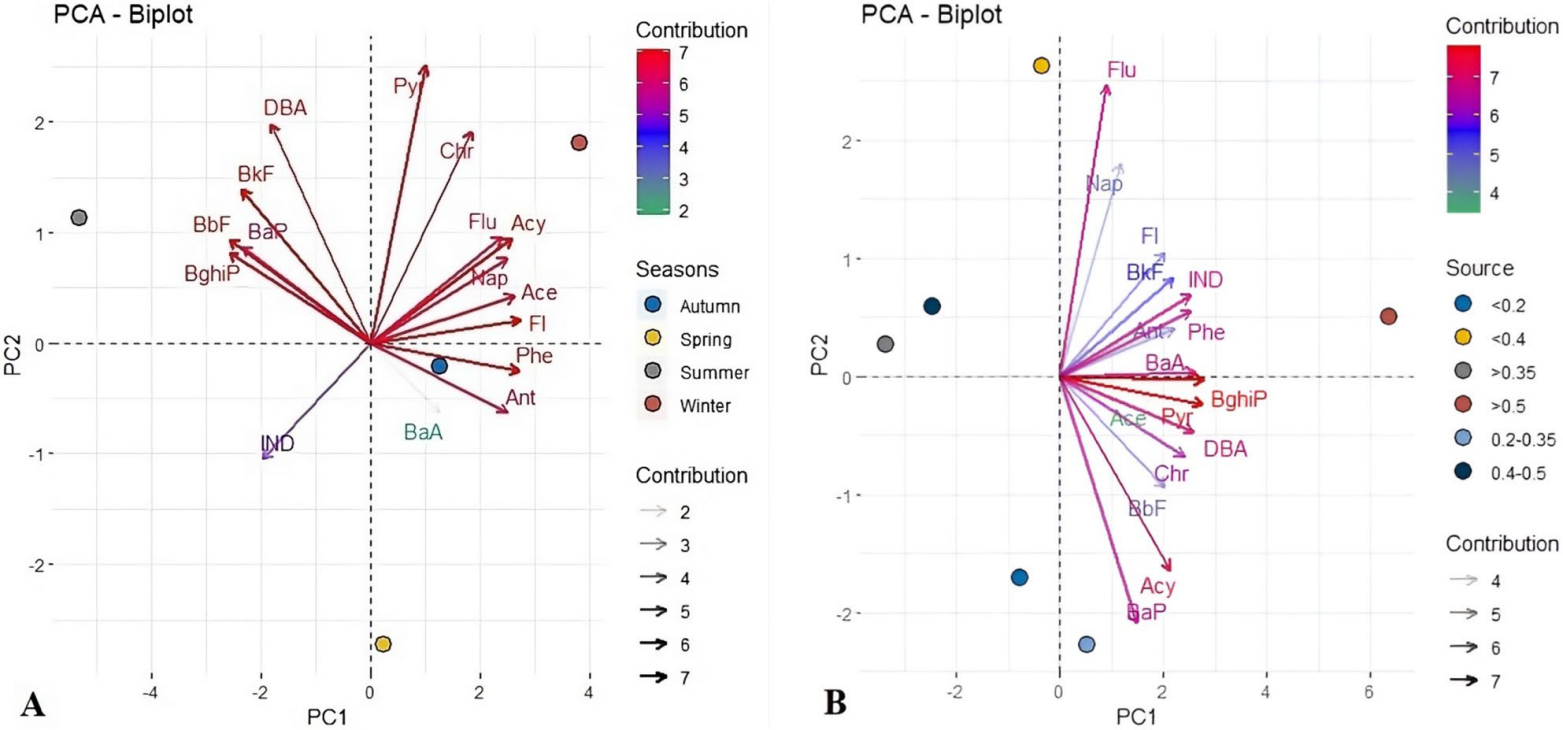
PCA analysis for water (**A**) and sediment (**B**).

PCA was used to describe the individual loading of 16 PAHs variables in water and sediment samples from all six sites in the Danube River (Fig. 7, plotted by R-Studio software). Bartlett's test revealed that the variables are substantially connected and appropriate for PCA analysis. The first two key components account for 88.6% of the overall variation in the set of findings related to water samples and 84.5% of the variance in the sediment samples. Generally, PCA1 was favourably dominated by high loadings of all examined PAHs in both water (Fig. 7A) and sediments (Fig. 7B). PCA analysis results corroborated with the previously observed distinction between sampling seasons, which were the concentrations of low molecular weight PAHs (Nap, Acy, Ace, Fl, Phe, and Ant) in water samples that were very high in cold seasons compared to the hot season. Specifically, from Fig. 7, it can be seen that according to PCA results, all the low molecular weight PAHs were characterized by cold seasons, which are winter, autumn, and spring. Contrarily, most of the high molecular weight PAHs occurred mainly in the summertime. As explained previously, this is attributed to poor water solubility and dissolution rate of HMWPAHs, which render them more resistant to decomposition, while LMWPAHs are more soluble and degradable during the hot season. It can be observed from Fig. 7 that the PCA results for sediment samples indicated that no different trends had been found for PAHs in seasonal sediment samples, demonstrating that sediments are independent of seasonal variations.

Table S6 displayed the concentrations range and toxicity recommendations for 16 individual PAHs. The concentrations of all PAHs in sediments were less than ERL except for Acy and FI concentrations, indicating the possibility of rare biological effects. However, the concentrations of both Acy in winter and spring and FI in autumn and spring were above ERL and lower than ERM, suggesting occasional biological effects. Generally, except for the two mentioned PAHs concentrations, eco-toxicological concerns for the aquatic environment of the Danube River do not pose a significant hazard. The combined impact of the 16 PAHs pollutants in sediments suggests a low chance for negative biological impacts and a low ecological threat. To guarantee that the residual levels of PAHs in the sediments of the Danube River do not surpass the ecological quality criteria; routine monitoring of PAHs in sediments is required. In addition, initiatives for pollution control must be undertaken to avoid the spread of PAHs in the Danube River.

**Figure 8:**
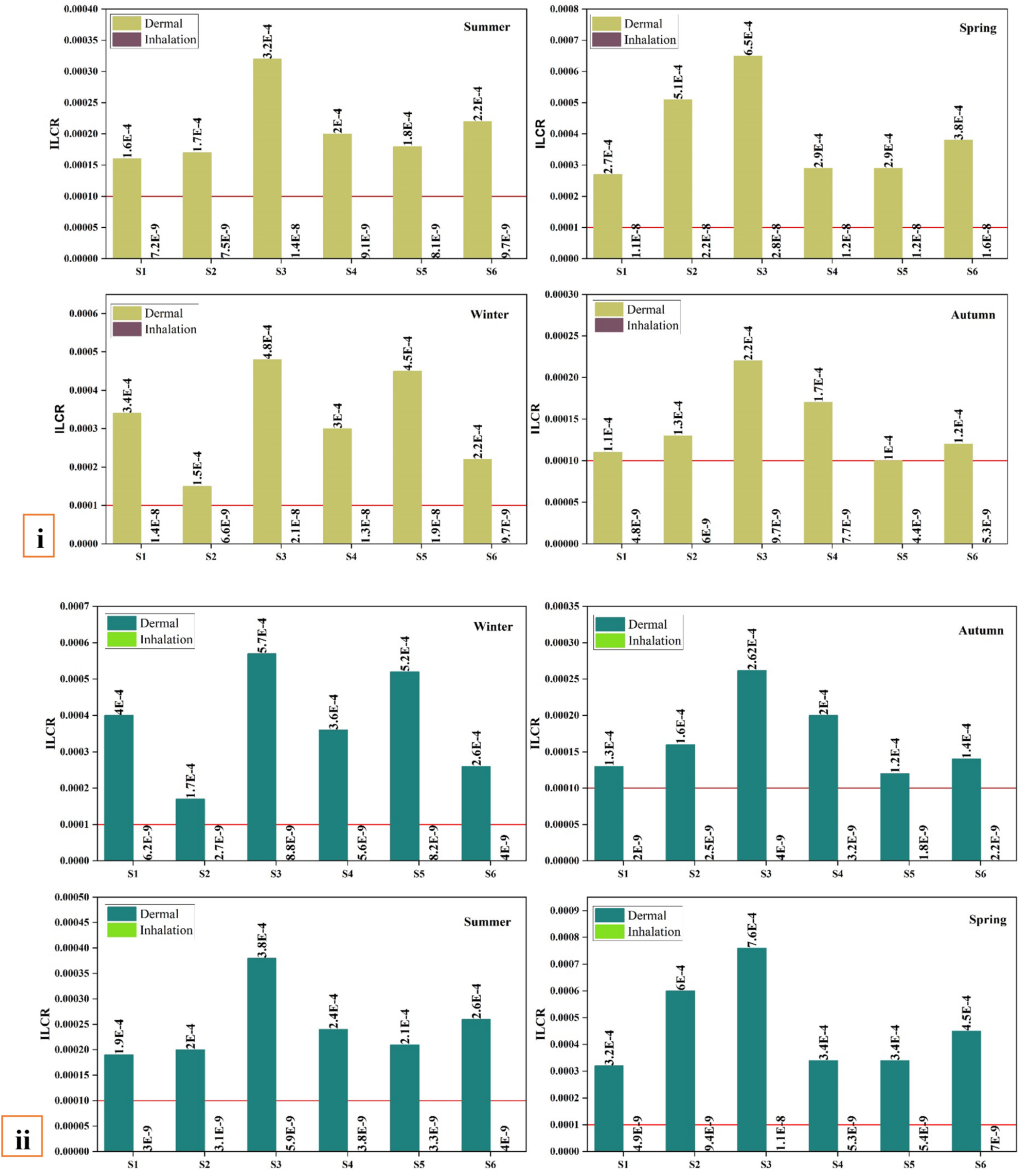
ILCR levels in the Danube River sediments for adults (**i**) and children (**ii**). The red line is the Incremental Lifetime Cancer Risk (ILCR) (1/10^4^).

The TEQ corresponds to sediment samples ranging from 29.88 in the winter season ng/g to 140.39 ng/g in the autumn season. According to the Canadian soil quality guidelines for the preservation of the ecosystem and human health, the threshold value of 600 ng/g is considered safe for humans^71^. For sediment samples, the ILCR values for adults and children detected in sediment samples from the Danube River are shown in Fig. 8. The total ILCR in both children and adults are more than 1/10^4^ in all seasons, with the highest values recorded in site 3, which is really a matter of serious concern. In addition, these records are substantially larger than those reported in the Brisbane River in Australia^72^. When high amounts are found through long-term surveillance, the residents within the river basin area must be warned, and precautions must be taken to prevent human contact with sediments.

## Conclusions

The current work provides a detailed evaluation of PAHs concentrations, seasonal distribution, and ecological risk assessment in water and sediments gathered from six distinct sites along the Danube River in Hungary. Temporal and spatial variations of PAHs were investigated in both rivers' water and sediments, reflecting the anthropogenic sources along the Danube River. The findings highlighted a broad variance range of 16PAHs contents in water with total concentrations of PAHs ranging from 283.1–541.9, 198.6–404.3, 160.0–324.6, and 228.2–378.1 ng/L for winter, spring, summer, and autumn, respectively. Sediment samples showed PAH ranging from 313.7–622.7, 312.7–595.4, 215.1–465.4, and 311.3–491.7 ng/g for winter, spring, summer, and autumn, respectively. The overall analysis of the results indicates that the putative anthropogenic sources of PAHs were verified to be both pyrogenic (incomplete combustion of biomass and coal) and pyrolytic (incomplete combustion of liquid fossil fuels and vehicle exhaust emissions); with pyrogenic origins predominating over pyrolytic sources. This might suggest that the industries essentially utilize fossil fuels, which would increase the PAHs emissions in the study area. Generally, except for Acy and FI concentrations, the eco-toxicological assessment of the Danube River environment showed no significant PAHs pollutants in sediments, suggesting a low chance for negative biological impacts and low ecological risk. The total ILCR in both children and adults were calculated to be more than 1/10^4^ in all seasons, with the highest values recorded in spring and followed by winter, which constitutes a concerning issue. Continuous monitoring of the PAHs would offer better insight into the scale of the pollution, which would help in devising effective mitigation strategies.

### Supplementary Information

Supplementary Information.

## Data Availability

The datasets used and/or analysed during the current study are available from the corresponding author upon reasonable request.

## Code availability

Codes are publicly available.
