# Supplementary material for: Temporal and spatial distribution of polycyclic aromatic hydrocarbons (PAHs) in the Danube River in Hungary
Source: Sci Rep. 2024 Apr 9;14:8318. doi: 10.1038/s41598-024-58793-2 (PMC11004153; doi:10.1038/s41598-024-58793-2)
Supplement: Supplementary file 1 — Supplementary Information. [file 41598_2024_58793_MOESM1_ESM.docx]

**Temporal and spatial distribution of Polycyclic Aromatic Hydrocarbons (PAHs) in the Danube River in Hungary**

**Ruqayah Ali Grmasha ^a,b,c^, Csilla Stenger-Kovács ^a,d^, Osamah J. Al-sareji ^b,c,^, Raed A. Al-Juboori ^e,f^, Mónika Meiczinger ^c^, Manolia Andredaki ^g^, Ibijoke A. Idowu ^g^, Hasan Sh. Majdi ^h^, Khalid Hashim ^g,b,j*^, Nadhir Al-Ansari ^k,*^**

^a^ Limnology Research Group, Center for Natural Science, University of Pannonia, Egyetem utca 10, H-8200, Veszprém, Hungary

^b^ Environmental Research and Studies Center, University of Babylon, Al-Hillah 51001, Iraq

^c^ Sustainability Solutions Research Lab, Faculty of Engineering, University of Pannonia, Egyetem str. 10, Veszprém, 8200, Hungary

^d^ HUN-REN–PE Limnoecology Research Group, Egyetem utca 10, H-8200, Veszprém, Hungary

^e^ NYUAD Water Research Center, New York University-Abu Dhabi Campus, Abu Dhabi, PO Box 129188, Abu Dhabi, United Arab Emirates

^f^ Water and Environmental Engineering Research Group, Department of Built Environment, Aalto University, PO Box 15200, Aalto, FI-00076, Espoo, Finland

^g^ School of Civil Engineering and Built Environment, Liverpool John Moores University, Liverpool, UK

^h^ Department of Chemical Engineering and Petroleum Industries, Al‐Mustaqbal University College, Hillah, Iraq.

^j^ Dijlah University College, Baghdad, Iraq

^k^ Department of Civil, Environmental and Natural Resources Engineering, Lulea University of Technology, Sweden.

*Corresponding author: nadhir.alansari@ltu.se, k.s.hashim@ljmu.ac.uk

| **Table S1** Sampling coordinates | | |
| --- | --- | --- |
| Sites | Latitude (N) | Longitude (E) |
| S1 | 47.788016 | 19.114344 |
| S2 | 47.680858 | 19.125760 |
| S3 | 47.523438 | 19.049630 |
| S4 | 47.248683 | 18.910842 |
| S5 | 46.912084 | 18.964844 |
| S6 | 46.616233 | 18.858203 |

| Table S2: The 16 PAHs with their abbreviations and specifications | | | | | |  |
| --- | --- | --- | --- | --- | --- | --- |
| Abb. | PAH | Molecular weight | CAS# | Ring | MW | TEF* |
| Nap | Naphthalene | LMW | 91-20-3 | 2 | 128.17 | 0.001 |
| Acy | Ascenaphthylene | LMW | 208-96-8 | 3 | 152.19 | 0.001 |
| Ace | Acenaphthene | LMW | 83-32-9 | 3 | 154.21 | 0.001 |
| Fl | Fluorene | LMW | 86-73-7 | 3 | 166.22 | 0.001 |
| Phe | Phenanthrene | LMW | 85-01-8 | 3 | 178.23 | 0.001 |
| Ant | Anthracene | LMW | 120-12-7 | 3 | 178.23 | 0.01 |
| Flu | Fluoranthene | HMW | 206-44-0 | 4 | 202.26 | 0.001 |
| Pyr | Pyrene | HMW | 129-00-0 | 4 | 202.26 | 0.001 |
| BaA | Benz(a)anthracene | HMW | 56-55-3 | 4 | 228.29 | 0.1 |
| Chr | Chrysene | HMW | 218-01-9 | 4 | 228.29 | 0.01 |
| BbF | Benzo(b)f luoranthene | HMW | 205-99-2 | 5 | 253.32 | 0.1 |
| BkF | Benzo(k)f luoranthene | HMW | 207-08-9 | 5 | 253.32 | 0.1 |
| BaP | Benzo(a)pyrene | HMW | 50-32-8 | 5 | 253.32 | 1 |
| DBA | Dibenz(a,h)anthracene | HMW | 215-58-7 | 6 | 278.35 | 1 |
| BghiP | Benzo (g,h,i) perylene | HMW | 191-24-2 | 6 | 276.34 | 0.01 |
| IND | Indeno(1,2,3-cd)pyrene | HMW | 193-39-5 | 6 | 276.34 | 0.1 |
| * [1]. | | | | | | |

| **Table S3**: Parameters descriptions used for ILCR model | | | | | | |
| --- | --- | --- | --- | --- | --- | --- |
| **Parameter** | **Description** | **Unit** | **Adults** | **Children** | | **References** |
| **ABS** | Dermal-Absorption-Factor | unitless | 0.13 | 0.13 | [2] | |
| **AF** | Dermal-Adherence-Factor | mg/cm^2^ | 0.07 | 0.2 | [2] | |
| **AT** | Average-Time (70years_365 days/year) | Days | 25,550 | 25,550 | [3] | |
| **BW** | Body-Weight | Kg | 70 | 15 | [4] | |
| **ED** | Exposure-Duration | Years | 20 | 6 | [4] | |
| **EF** | Exposure-Frequency | days/year | 350 | 350 | [4] | |
| **IR ingestion** | Ingestion-Rate | mg/day | 100 | 200 | [2] | |
| **IR inhalation** | Inhalation-Rate | m^3^/day | 20 | 10 | [3] | |
| **PEF** | Particular-Emission-Factor | m^3^/kg | 1.36×10^9^ | 1.36×10^9^ | [4] | |
| **SA** | Dermal-Surface-Area-Exposure | cm^2^ | 5700 | 2800 | [4] | |
|  |  |  |  |  |  | |

| **Table S4.** Sources diagnostic ratios of PAHs in water Explanation of PAHs diagnostic ratios | | | | |
| --- | --- | --- | --- | --- |
| **Ratio** | **Range** | **Source** | **Calculated Ratios** | **References** |
| LMW/HMW | >1 | Petrogenic inputs (liquid fuel discharges) | Winter = 2.26 > 1  Spring = 1.70 > 1  Summer = 0.48 < 1  autumn= 1.60 >1 | [5,6] |
|  | <1 | Pyrogenic - Combustion of solid fuel - natural sources such as biomass (grass and wood) and coal |  |  |
| Flu/(Flu+Pyr) | <0.4 | Petrogenic inputs (liquid fuel discharges) | Winter = 0.42 ( 0.4-0.5)  Spring = 0.49 ( 0.4-0.5)  Summer = 0.36 < 0.4  Autumn = 0.45 ( 0.4-0.5) | [5, 7, 8] |
|  | 0.4-0.5 | Pyrolytic (burning of liquid fossil fuels and crude oil. vehicles) |  |  |
|  | >0.5 | Pyrogenic - Combustion of solid fuel - natural sources such as biomass (grass and wood) and coal |  |  |
| IND/(IND+BghiP) | <0.2 | Petrogenic inputs (liquid fuel discharges) | Winter = 0.48 ( 0.2-0.5)  Spring = 0.58 > 0.5  Summer = 0.35 (0.2-0.5)  Autumn = 0.41 ( 0.2-0.5) | [5, 7, 8] |
|  | 0.2-0.5 | Pyrolytic (burning of liquid fossil fuels and crude oil. vehicles) |  |  |
|  | >0.5 | Pyrogenic - Combustion of solid fuel - natural sources such as biomass (grass and wood) and coal |  |  |
| BaA/(BaA+Chr) | <0.2 | Petrogenic inputs (liquid fuel discharges) | Winter = 0.37 > 0.35  Spring = 0.45 > 0.35  Summer = 0.44 > 0.35  Autumn = 0.48 > 0.35 | [5, 7, 8] |
|  | 0.2-0.35 | Mixed sources (petrogenic / pyrogenic) |  |  |
|  | >0.35 | Pyrogenic - Combustion of solid fuel - natural sources such as biomass (grass and wood) and coal |  |  |
| BaP/(BaP+Chr) | <0.4 | Petrogenic inputs (liquid fuel discharges) | Winter = 0.35 < 0.4  Spring = 0.49 > 0.4  Summer = 0.62 > 0.4  Autumn = 0.55 > 0.4 | [9] |
|  | >0.4 | Pyrogenic - Combustion of solid fuel - natural sources such as biomass (grass and wood) and coal |  |  |
| BaP/BghiP | >0.6 | Petrogenic inputs (liquid fuel discharges) | Winter = 0.95 > 0.6  Spring = 0.78 > 0.6  Summer = 0.68 > 0.6  Autumn = 1.19 >0.6 | [10] |
|  | <0.6 (non-traffic emission) | Pyrogenic - Combustion of solid fuel - natural sources such as biomass (grass and wood) and coal |  |  |

| **Table S5**Sources diagnostic ratios of PAHs in sediments Explanation of PAHs diagnostic ratios | | | | |
| --- | --- | --- | --- | --- |
| Ratio | Range | Source | Calculated Ratios | References |
| LMW/HMW | >1 | Petrogenic inputs (liquid fuel discharges) | Winter = 0.34 < 1  Spring = 0.43 < 1  Summer = 0.39 < 1  autumn= 0.39 < 1 | [5,6] |
|  | <1 | Pyrogenic - Combustion of solid fuel - natural sources such as biomass (grass and wood) and coal |  |  |
| Flu/(Flu+Pyr) | <0.4 | Petrogenic inputs (liquid fuel discharges) | Winter = 0.32 < 0.4  Spring = 0.37 < 0.4  Summer =0.43 (0.4-0.5)  Autumn = 0.54 > 0.5 | [5, 7, 8] |
|  | 0.4-0.5 | Pyrolytic (burning of liquid fossil fuels and crude oil. vehicles) |  |  |
|  | > 0.5 | Pyrogenic - Combustion of solid fuel - natural sources such as biomass (grass and wood) and coal |  |  |
| IND/(IND+BghiP) | <0.2 | Petrogenic inputs (liquid fuel discharges) | Winter = 0.53 > 0.5  Spring = 0.37 (0.2-0.5)  Summer = 0.51 > 0.5  Autumn = 0.57 > 0.5 | (7; 5;8) |
|  | 0.2-0.5 | Pyrolytic (burning of liquid fossil fuels and crude oil. vehicles) |  |  |
|  | >0.5 | Pyrogenic - Combustion of solid fuel - natural sources such as biomass (grass and wood) and coal |  |  |
| BaA/(BaA+Chr) | <0.2 | Petrogenic inputs (liquid fuel discharges) | Winter = 0.39 > 0.35  Spring = 0.51 > 0.35  Summer=0.28 (0.2-0.35)  Autumn = 0.55 > 0.35 | [5, 7, 8] |
|  | 0.2-0.35 | Mixed sources (petrogenic / pyrogenic) |  |  |
|  | >0.35 | Pyrogenic - Combustion of solid fuel - natural sources such as biomass (grass and wood) and coal |  |  |
| BaP/(BaP+Chr) | <0.4 | Petrogenic inputs (liquid fuel discharges) | Winter = 0.39 < 0.4  Spring = 0.68 > 0.4  Summer = 0.52 > 0.4  Autumn = 0.70 > 0.4 | [9] |
|  | >0.4 | Pyrogenic - Combustion of solid fuel - natural sources such as biomass (grass and wood) and coal |  |  |
| BaP/BghiP | > 0.6 | Petrogenic inputs (liquid fuel discharges) | Winter = 1.70 > 0.6  Spring = 1.1 > 0.6  Summer = 2.45 > 0.6  Autumn = 5.10 > 0.6 | [10] |
|  | < 0.6 (non-traffic emission) | Pyrogenic - Combustion of solid fuel - natural sources such as biomass (grass and wood) and coal |  |  |

| **Table S5:** Concentration range of 16 individual PAHs (ng/g dw) and toxicity guidelines | | | | | | |
| --- | --- | --- | --- | --- | --- | --- |
| PAHs | ERL-ERM | Range (ng/g dw) | | <ERL | ≥ERL and <ERM | ≥ERM |
|  |  | Min Max | |  |  |  |
| Nap | 160–2100 | 14.01 | 19.23 | ✓ |  |  |
| Acy | 16–500 | 11.67 | 18.45 | Rest Seasons | Winter, Spring |  |
| Ace | 44–640 | 11.20 | 21.99 | ✓ |  |  |
| FI | 19–540 | 11.89 | 23.30 | Rest Seasons | Spring autumn |  |
| Phe | 240–1500 | 14.58 | 23.21 | ✓ |  |  |
| Ant | 600–5100 | 13.21 | 24.85 | ✓ |  |  |
| Flu | 85.3–1100 | 8.99 | 21.44 | ✓ |  |  |
| Pyr | 665–2500 | 11.40 | 36.00 | ✓ |  |  |
| BaA | 261–1600 | 10.17 | 32.88 | ✓ |  |  |
| Chr | 384–2800 | 16.16 | 50.82 | ✓ |  |  |
| BbF | 320–1880 | 22.93 | 42.62 | ✓ |  |  |
| BkF | 280–1620 | 26.31 | 36.85 | ✓ |  |  |
| BaP | 430–1600 | 28.79 | 52.47 | ✓ |  |  |
| DBA | 63.4–260 | 14.97 | 42.42 | ✓ |  |  |
| BghiP | 430–1600 | 13.33 | 33.81 | ✓ |  |  |
| IND | 240- | 17.00 | 29.38 | ✓ |  |  |

**References**

**[1] Nisbet**, I.C. and Lagoy, P.K., 1992. Toxic equivalency factors (TEFs) for polycyclic aromatic hydrocarbons (PAHs). *Regulatory toxicology and pharmacology*, *16*(3), pp.290-300.

**[2] US EPA,** 2011. Exposure Factors Handbook. 2011 ed., Final Report. Environmental Protection Agency, Washington, DC EPA/600/R-09/052F.

**[3] Soltani,** N., Keshavarzi, B., Moore, F., Tavakol, T., Lahijanzadeh, A.R., Jaafarzadeh, N. and Kermani, M., 2015. Ecological and human health hazards of heavy metals and polycyclic aromatic hydrocarbons (PAHs) in road dust of Isfahan metropolis, Iran. Science of the total environment, 505, pp.712-723.

**[4] U.S.** Environmental Protection Agency, 2014. Human health evaluation manual, supplemental guidance: update of standard default exposure factors. OSWER Directive 9200 1–120.

**[5] Baran**, A., Klimkowicz-Pawlas, A., Ukalska-Jaruga, A., Mierzwa-Hersztek, M., Gondek, K., Szara-Bąk, M., Tarnawski, M. and Spałek, I., 2021. Distribution of polycyclic aromatic hydrocarbons (PAHs) in the bottom sediments of a dam reservoir, their interaction with organic matter and risk to benthic fauna. *Journal of Soils and Sediments*, *21*(6), pp.2418-2431.

**[6] Yang**, Y., Wang, H., Chang, Y., Yan, G., Chu, Z., Zhao, Z., Li, L., Li, Z. and Wu, T., 2020. Distributions, compositions, and ecological risk assessment of polycyclic aromatic hydrocarbons and phthalic acid esters in surface sediment of Songhua river, China. *Marine Pollution Bulletin*, *152*, p.110923.

**[7] Adeniji**, A.O., Okoh, O.O. and Okoh, A.I., 2019. Levels of polycyclic aromatic hydrocarbons in the water and sediment of Buffalo River Estuary, South Africa and their health risk assessment. *Archives of environmental contamination and toxicology*, *76*, pp.657-669.

**[8] Montuori**, P., Aurino, S., Garzonio, F., Sarnacchiaro, P., Nardone, A. and Triassi, M., 2016. Distribution, sources and ecological risk assessment of polycyclic aromatic hydrocarbons in water and sediments from Tiber River and estuary, Italy. *Science of the Total Environment*, *566*, pp.1254-1267.

**[9] Yunker**, M.B., Macdonald, R.W., Vingarzan, R., Mitchell, R.H., Goyette, D. and Sylvestre, S., 2002. PAHs in the Fraser River basin: a critical appraisal of PAH ratios as indicators of PAH source and composition. *Organic geochemistry*, *33*(4), pp.489-515.

**[10] Barhoumi**, B., Beldean-Galea, M.S., Al-Rawabdeh, A.M., Roba, C., Martonos, I.M., Bălc, R., Kahlaoui, M., Touil, S., Tedetti, M., Driss, M.R. and Baciu, C., 2019. Occurrence, distribution and ecological risk of trace metals and organic pollutants in surface sediments from a Southeastern European river (Someşu Mic River, Romania). *Science of the Total Environment*, *660*, pp.660-676.
